# Guiding warfarin self-management in children: development of a warfarin nomogram

**DOI:** 10.1016/j.rpth.2023.102255

**Published:** 2023-11-10

**Authors:** Sophie Jones, Jodi Hislop, Ashleigh Allan, Adele Kuzmitsky, Michael Pham-Crepps, Anthea Greenway, Paul Monagle, Fiona Newall

**Affiliations:** 1 The University of Melbourne, Department of Nursing, Melbourne, Australia; 2 Murdoch Children’s Research of Institute, Melbourne, Australia; 3 Department of Clinical Haematology, Royal Children’s Hospital, Melbourne, Australia; 4 Nursing Research Department, Royal Children’s Hospital, Melbourne, Australia; 5 University of Melbourne, Department of Paediatrics, Melbourne Australia

**Keywords:** anticoagulant, child, nomograms, self-management, warfarin

## Abstract

**Background:**

Warfarin therapy in children is impacted by many variables. To support the implementation of a self-management program within a pediatric anticoagulation service, a pediatric-specific warfarin nomogram was needed. A literature review revealed no published pediatric nomograms; therefore, a nomogram was developed drawing upon an evidence-based “Warfarin Information for Clinicians” hospital guideline.

**Objectives:**

This study aimed to evaluate the suitability of a pediatric warfarin nomogram.

**Methods:**

A retrospective audit of electronic medical records compared the dosing and international normalized ratio (INR) retest decisions made by hematology clinicians to the dosing and retesting recommended by a new warfarin nomogram at a pediatric hospital. Children (aged 6 months-18 years) receiving warfarin therapy for >6 months were included. Data were collected between September 2019 and February 2020. Descriptive data analysis was performed. The study was approved by the hospital’s Human Research Ethics Committee.

**Results:**

Warfarin dosing and INR retest decisions for 39 children were included, equating to 521 INRs. The nomogram matched 81.4% of clinicians dosing decisions and 30% of INR retest decisions. Moreover, 59% of the clinician-recommended retest dates were earlier than the nomogram recommendation. In the INR 2.0-3.0 group, 84.4% of dosing decisions and 72% of retest decisions matched the nomogram.

**Conclusions:**

These results suggest that this pediatric nomogram is a suitable tool for warfarin dosing, as recommended warfarin doses matched the majority of clinicians’ decisions. Modification may be needed to nomogram recommendations for the time to retest. This nomogram can be used to support warfarin self-management and may assist clinicians and patients or families in making evidence-based dosing decisions.

## Essentials

• To support a self-management program, a new pediatric warfarin nomogram was developed.
• The nomogram was compared with expert hematology clinician warfarin dosing.
• The pediatric nomogram is suitable as doses matched most clinicians’ recommendations.
• The nomogram supports evidence-based warfarin dosing for families undertaking self-management.

## 1 Introduction

Increasing numbers of pediatric patients with chronic conditions require thromboprophylaxis following greater surgical options with advances in medicine [1,2]. Warfarin is commonly prescribed to the pediatric population as long-term anticoagulation therapy, particularly following the Fontan procedure or placement of prosthetic heart valves [[3], [4], [5]]. To ensure the safety and effectiveness of warfarin, which has a narrow therapeutic index, close monitoring of children’s international normalized ratio (INR) and dose adjustments are necessary to avoid both iatrogenic bleeding and thrombotic complications [2,6,7]. The frequent testing and dose adjustments required during long-term warfarin therapy may become a chronic burden for children and their families, negatively impacting their health-related quality of life [8,9]. The management of long-term warfarin therapy is more complex in children than in the adult population due to their inconsistent nutritional intake, smaller physical size, complex health conditions, lifestyle, and higher infection rates [4,10,11].

The introduction of specialized pediatric anticoagulation clinics has led to an increased focus on supporting families to take a greater role in their child’s warfarin therapy through patient self-testing and patient self-management (PSM) [1,12]. In patient self-testing, a point-of-care device, such as the CoaguChek XS (Roche Diagnostics), is used by families to determine the child’s INR through a sample of the child’s capillary blood [10,[13], [14], [15], [16]]. This result is then reported to health services so that hematology clinicians can advise the family of any warfarin dose adjustments required and when to retest the child’s INR [4,17]. The safety, efficacy, and validity of this INR testing method have been confirmed for both adults and pediatric warfarin patients [10,[13], [14], [15], [16]].

Pediatric warfarin PSM involves families testing the child’s INR with a point-of-care device and then, if required, altering the dose of warfarin administered to maintain the child’s INR within their target therapeutic range (TTR) [[18], [19], [20]]. Several small pediatric studies have demonstrated that empowering families to undertake PSM is safe, increases their knowledge of the child’s condition, and improves their commitment to the child’s health care, warfarin therapy adherence, and health-related quality of life [[18], [19], [20]]. Two previous PSM studies reported the dosing strategy used to guide families undertaking warfarin PSM, but no studies have validated the dosing strategy or tool utilized by families to make warfarin dosing and INR retesting decisions [14,18].

To support the implementation of a self-management program within a pediatric anticoagulation service, a pediatric-specific warfarin nomogram was needed. Outcomes of the warfarin self-management program have been reported separately [21]. The primary objective of this study was to determine the suitability of a “new” pediatric warfarin nomogram as a tool for guiding dosing and INR retesting decisions for children receiving long-term warfarin therapy.

## 2 Methods

### 2.1 Study design and setting

A single-center retrospective audit was conducted at a tertiary pediatric hospital to evaluate the suitability of a new pediatric warfarin nomogram. This was achieved by comparing the warfarin dosing and INR retesting decisions made by hematology clinicians, using the hospital’s guidelines and their clinical expertise, to the recommendations of the new pediatric warfarin nomogram. The audit period was from the September 1, 2019, to February 29, 2020 (inclusive). Ethics approval was obtained from the hospital’s Human Research Ethics Committee (approval number QA/60343/RCHM-2020).

### 2.2 Pediatric warfarin nomogram

A novel pediatric warfarin nomogram was developed to support a PSM program based on the institutional Warfarin Information for Clinicians evidence-based guideline [21,22]. The nomogram was developed with expert input from a pediatric hematologist (P.M.) and anticoagulation clinical nurse consultants (S.J., F.N.) managing pediatric warfarin dosing daily. A different nomogram was developed for each target therapeutic INR range which provides dose recommendation, factors to consider that may have influenced INR results, and recommends a time to retest the INR. The nomogram was to be used in combination with guidance documents that detail the impact of known factors on warfarin dose requirements and INR results. Users of the nomogram are requested to consider the trend of the INRs and the stability of the INR over time. The nomogram has been developed to encourage users to problem-solve based on the child’s individual dose requirements and clinical status, rather than following a prescriptive dosing regimen merely based on the INR result that day. This is presented to user as a two-step process; step 1: ask users to follow a table to determine INR stability and consider previous INR results; step 2: then provides guidance to users on the appropriate dose (change or remain the same), depending on stability and whether the INR is in range that day. The table for step 1 (INR stability) and step 2 (nomogram) for target INR range of 2.0 to 3.0 has been provided as supporting information.

### 2.3 Study population

Pediatric patients who were receiving long-term warfarin therapy, being managed by the Clinical Hematology department and meeting the inclusion criteria were eligible. Inclusion criteria were patients aged from 6 months to 18 years of age; who have received warfarin therapy for at least 6 months; who are not self-managing their warfarin therapy; who have not had an interruption to their warfarin therapy during the audit period due to the difficulty in obtaining therapeutic INRs after a period of cessation. Patients meeting the inclusion criteria for the study were identified from an existing database of all warfarin patients held by the Clinical Haematology department. The decision to exclude patients on warfarin therapy for <6 months was due to the instability of the patient’s INR during the first 6 months of warfarin therapy [23]. Participants included in the study were selected through purposive sampling to ensure an even spread of TTR and sufficient warfarin dosing decisions across the cohort. A sample size of 50 patients was deemed feasible for the purpose and scale of this study, expecting that each participant will contribute multiple INRs for the audit period and each INR represents a data point.

### 2.4 Data collection

Data were retrieved retrospectively from the hospital electronic medical record, Epic, in March 2020. Data were entered as deidentified data into a password-protected Microsoft Excel (Version 1908, 2022, Washington) spreadsheet. Information obtained from Epic included participants’ age, indication for warfarin therapy, time on warfarin therapy, and target INR range. For the audit period, each recorded INR value, warfarin dosing, and INR retesting decision made by a clinician were recorded. Factors contributing to INR instability and dosing decisions, such as medication changes, diet changes, missed warfarin doses, illness, or growth as documented in Epic were also recorded. These data were used to determine the level of agreement between the dosing decision made by the hematology clinicians and the recommendations of the new nomogram. Random cross-checking of 10% of the data collected was conducted to monitor its accuracy and determine interrater reliability among data collectors.

The incidence of any thrombotic or bleeding complications was recorded through examination of dosing comments and clinician notes. Bleeding is defined as major, clinically relevant bleeding, or minor. Major bleeding is defined as “composite of fatal bleeding; clinically overt bleeding associated with a fall in hemoglobin level of ≥20 g/L in a 24 hour period; bleeding that is retroperitoneal, pulmonary, intracranial, or involves the central nervous system; and bleeding that requires surgical intervention in an operating suite” [24] (p. 1857). Clinically relevant non–major bleeding is defined as a composite of overt bleeding requiring a blood transfusion which “is not directly attributable to the patient’s underlying medical condition and bleeding that requires medical or surgical intervention to restore hemostasis, other than in an operating suite” [24]. Minor bleeding is defined as any overt or macroscopic bleeding that does not meet the definitions for major or clinically relevant bleeding [24].

### 2.5 Outcome measures

A level of agreement of >75% was expected between the clinician decisions and the recommendation of the new nomogram (warfarin dose and INR retest date) to deem the nomogram reliable for use in supporting PSM. The level of agreement was based on standard accepted level of interrater reliability for decision support tools [25,26]. Comparison of the warfarin dosing and INR retest date recommended by the hematology clinicians and by the nomogram were reported for the cohort as a whole and for each TTR. This was performed as the nomogram has separate components specific to each TTR.

### 2.6 Statistical analysis

Data were analyzed descriptively in in Microsoft Excel (version 1908, 2022) and Statistical Package for Social Science (version 27, 2020, IBM) to determine the interrater reliability of warfarin dosing and INR retesting decisions between the clinicians and the nomogram. Interrater reliability of dosing and retest decisions were measured by percentage agreement, calculated as the number of agreeing observations divided by the total number of observations. The percentage of INRs within participants’ TTR was presented using cluster analysis [16,27] and described using medians (with ranges) as appropriate. Bleeding events were reported descriptively.

## 3 Results

Of 202 children requiring warfarin at our institution, 139 were eligible and met the inclusion criteria. Fifty children were purposively selected to provide a representative sample. Data collection was haltered early as the COVID-19 pandemic began, and the study team was not allowed on site. The hospital denied team members responsible for data collection remote access to the electronic medical records to continue data collection. Data were ultimately collected for 39 children who satisfied the inclusion criteria. Table 1 presents the indications for warfarin for the children included in the study and for those excluded (despite being eligible).

**Table 1:** Indication for warfarin.

Indication	Participants (*n* = 39), number (%)	Excluded (*n* = 100), number (%)
Fontan procedure	21 (53.8)	82 (82)
Prosthetic valve	8 (20.5)	5 (5)
Central venous catheter	5 (12.8)	2 (2)
Kawasaki disease	3 (7.7)	6 (6)
Other	2 (5.1)	5 (5)

The median age of the children was 10.6 years (range, 2.1-18.4 years), and children had been receiving warfarin for a median of 5.5 years (range, 0.5-14.9 years). The most common indications for warfarin therapy were a previous Fontan procedure (*n* = 20) and the presence of a prosthetic heart valve (*n* = 8). The 39 children included in the study contributed 521 INR tests, with a median of 12 tests per participant (range, 2-50). The greatest number of INR tests were analyzed in the 2.0-3.0 TTR (*n* = 303), followed by the 2.5 to 2.5 TTR (*n* = 105) and 3.0 to 4.0 TTR (*n* = 85). Missing data resulted in 517 clinician warfarin dosing decisions and 441 clinician-recommended INR retests dates that could be compared with the recommendations of the new pediatric warfarin nomogram. Table 2 summarizes participant’s target therapeutic ranges and TTR achievement.

**Table 2:** Target therapeutic range, time in therapeutic range achievement and agreement between clinician and nomogram recommendations.

Variable	Participants (*n* = 39)	
Number of INR tests per patient, median (range)	12 (2-50)	
Target therapeutic range, number (%)		
1.5-2.0	2 (5.1)	
2.0-2.5	1 (2.6)	
2.0-3.0	26 (66.7)	
2.5-3.5	7 (17.9)	
3.5-4.0	3 (7.7)	
	*N*	*n* (%)
Percentage of all INRs in therapeutic range	521	319 (61.2%)
Time in therapeutic range per patient, median % (range)^a^	38	65.9% (30%-100%)
Nomogram matched clinician dosing	517	421 (81.4%)
Nomogram recommended time of next INR matched clinician recommendation, number (%)	441	132 (29.9%)
Nomogram recommended time of next INR earlier/later than clinician recommendation, number (%)	Earlier	48 (10.9)
	Later	261 (59.2)

a Cluster analysis per patient. INR, international normalized ratio.

### 3.1 Warfarin dosing decisions

**Figure 1:**
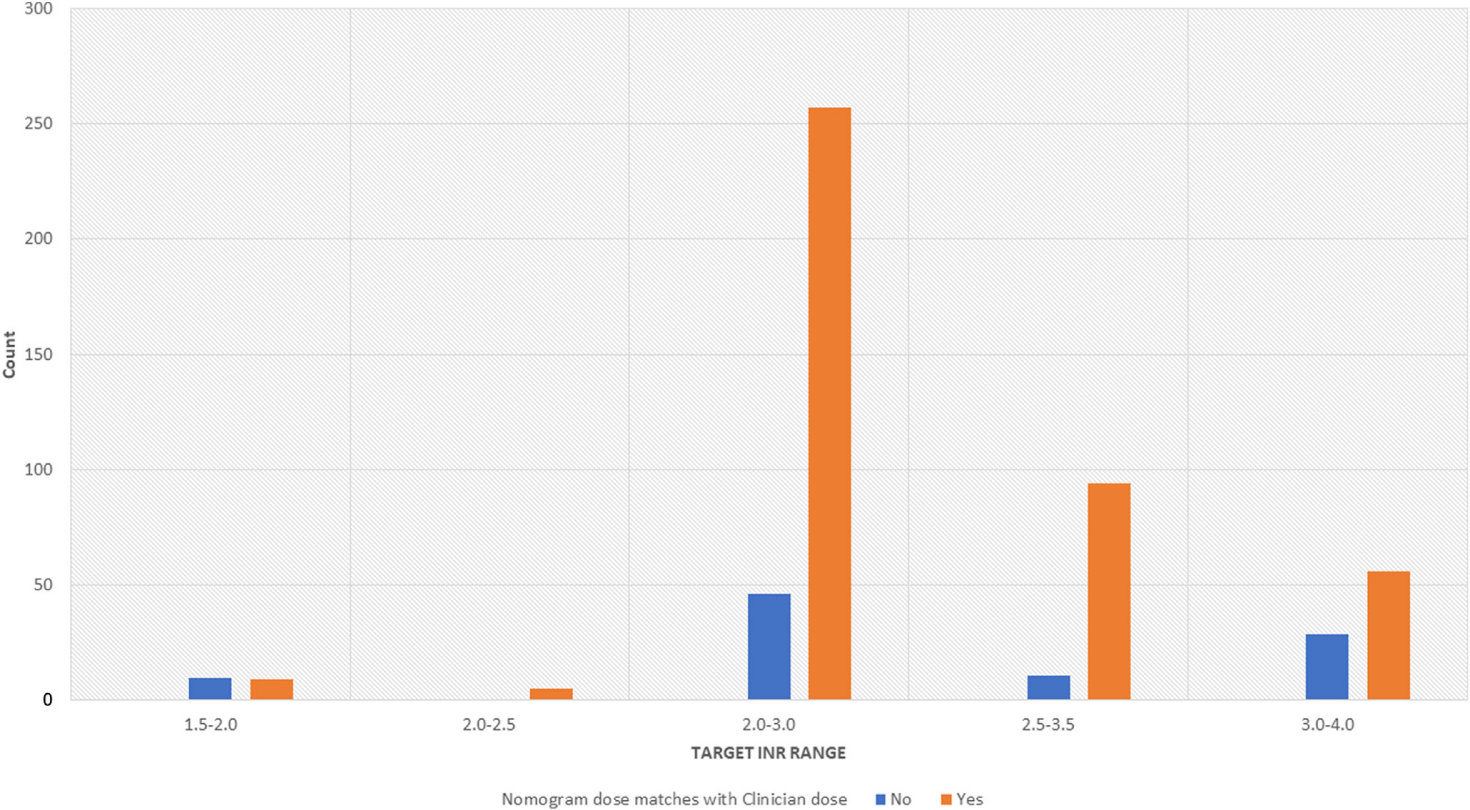
Agreement between clinician dose and nomogram dose by target international normalized ratio range.

Across the cohort, the majority (81.4%, *n* = 421) of warfarin dosing decisions made by the hematology clinicians matched the doses recommended by pediatric warfarin nomogram. Figure 1 presents the percentage of warfarin doses recommended by the nomogram in each TTR that matched the hematology clinician’s dosing decisions. The highest percentage of matches were obtained in the 2.0 to 3.0 TTR (*n* = 257, 84.8%).

### 3.2 Recommended date of next INR test

**Figure 2:**
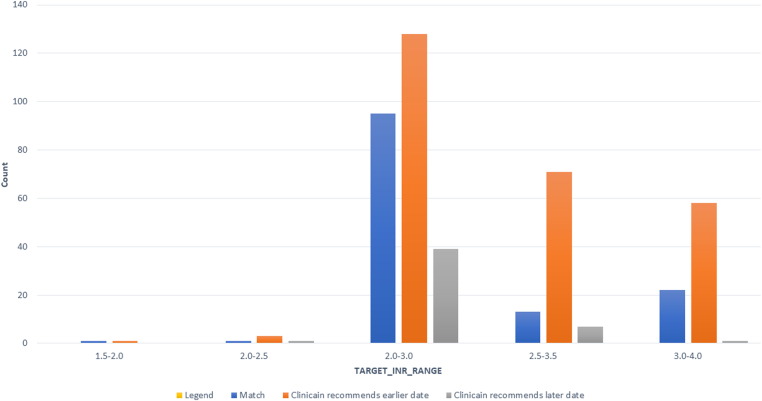
Agreement between clinician recommended date of international normalized ratio retest and nomogram recommendation by target international normalized ratio range.

Hematology clinicians recommended an INR retest date that was earlier than the nomogram recommendation in 59.2% of the 441 INR retest dates analyzed. The retest date recommended by the nomogram most accurately matched the retest decisions made by hematology clinicians for patients with at TTR of 2.0 to 3.0 (72%). Figure 2 presents the percentage of INR retest dates made by the nomogram that match the recommendations of hematology clinicians and the percentage that were earlier or later.

### 3.3 Percentage of INR tests within participants’ TTR

The nomogram recommendation matched to more clinician’s warfarin dosing and INR retest recommendations when participants had a greater percentage of their INR test results within their TTR. This is demonstrated in Table 3. For participants who had 75% or greater of their INR test within their TTR, a median of 100% of dosing decisions and 39% of INR retest recommendations recorded by hematology clinicians matched the recommendations of the nomogram.

**Table 3:** INR tests matched between clinician and nomogram for percentage of time in therapeutic range achievement.

Tests in TTR (%)	Participants^a^ total INR tests (*N*)	Matched dosing decisions, median (range) (%)	Matched INR retest date, median (range) (%)
0-50	159 (9)	88.9 (44-100)	22 (0-47)
50-74.9	215 (14)	81.5 (54-100)	39 (0-66)
75-100	155 (15)	100 (77-100)	39 (0-100)

a One participant with a single warfarin dosing decision and no INR retest recommendations were excluded. INR, international normalized ratio; TTR, target therapeutic range.

### 3.4 Supratherapeutic INRs

There were 11 instances when a child had an INR ≥5.0. The majority of INRs >5.0 were reported in children with a TTR of 2.5 to 3.5 (*n* = 3, 27.3%) or 3.0 to 4.0 (*n* = 6, 54.5%). In all but 3 instances, the dose recommended by the clinician matched the nomogram and the dose was either withheld that day or reduced significantly. There was one minor bleed, a brief episode of epistaxis, associated with an INR greater than 5.0. There were no thrombotic complications for any patients during the study period.

## 4 Discussion

This study aimed to determine the suitability of a pediatric warfarin nomogram for guiding warfarin dosing and INR retesting recommendations for children on long-term warfarin therapy undertaking PSM.

The median time in therapeutic range based on cluster analysis for the 39 children was 65.9%. For children who had >75% of their INR test results within their TTR (*n* = 15), the nomogram matched a median of 100% of dosing decisions made by clinicians. Twelve of the 15 children with >75% of their INR test results in target range had a TTR of 2.0 to 3.0. This suggests that dosing when the INR is in TTR is a predictable decision and that the TTR of 2.0 to 3.0 is easier to maintain target range achievement. This result follows previous pediatric studies investigating the safety and efficacy of warfarin management that report lower target range achievement for children with higher TTRs, such as 2.5 to 3.5 or 3.0 to 4.0 [28,29]. Children with higher TTRs, such as those with mechanical heart valves, are also more likely to need more frequent INRs and dose changes [28,29]. This can be due to clinician anxiety associated with the risk of thrombosis or bleeding with sub/supratherapeutic INRs in this population with a high TTR [28].

The study demonstrates that the new nomogram is suitable for warfarin dosing, as 81.4% of the 517 dosing decisions warfarin doses recommended by the nomogram matched the expert dosing decisions of hematology clinicians within a dedicated anticoagulation service. The level of agreement between the new nomogram and hematology clinicians’ dosing decisions was highest when compared for children with a TTR of 2.0 to 3.0.

There was less agreement between the nomogram and clinician decisions for the INR retest date. The nomogram recommended a longer interval between INR tests compared with the hematology clinician’s recommendation for 59.2% of INRs analyzed. Clinicians recommended a retest date earlier than the nomogram for the majority of the INRs analyzed. This could be due to caution on behalf of the clinicians or may be indicative of the expertise with warfarin dosing among the clinical team. The nomogram recommends that when the INR is in range and there have been no changes to the child’s clinical status, the time to the next test is 2 weeks, and then 4 weeks after 2 consecutive therapeutic INRs. Previous studies have routinely suggested the average time between INRs for most children is 2 weeks [2,4,30,31]. The option for families to perform a self-test at home means that frequency of INR testing can increase with minimal disruption to the family [32,33]. However, frequency of testing must be balanced with the costs associated with a home INR test ($AUD 13.06 per test) [34] and the clinical indication to test. Testing the INR too frequently may distort the true trajectory of the patient’s warfarin stability due to the long half-life of vitamin K antagonists and individual children’s responses to dose changes [35]. Due to the many factors affecting warfarin stability in children, adult recommendations for 4 weekly INR testing are often not appropriate [32,33]. Thus, retesting according to any changes in a child’s clinical status or a sudden drop or increase in the INR seems sensible.

Given the current lack of research into the use of pediatric warfarin nomograms, the information provided by this study is valuable and confirms that a nomogram is suitable for warfarin dose adjustment, after appropriate education, in children undertaking PSM or families dosing with support from an anticoagulation service. Although this study is the first to focus on the suitability of a pediatric warfarin nomogram, 2 pediatric studies have reported on the use of warfarin algorithms to guide families undertaking PSM to make warfarin dosing and INR retesting decisions [14,18]. A pilot study by Bauman et al. (2010) [14], determined that families (*N* = 14) performing PSM using a standardized warfarin dosing algorithm made warfarin dosing and INR retesting decisions that were consistent with guidelines in 90% of instances. Although the percentage of warfarin dosing and INR retesting decisions that matched the guidelines were not reported separately, the proportion of INR retest dates that matched is higher than that reported in our study [14]. Consistent with our findings, Bauman et al. [2] identified that the algorithm recommended a later INR retest date compared with when families retested the INR [2]. The ability of families to use this new pediatric warfarin nomogram to determine warfarin dosing and INR retesting recommendations for self-management has been evaluated [21]. The nomogram was found to be easy to follow and helpful for families to make decisions both when the INR was in and out of their child’s TTR [21].

While the nomogram did not always align directly with the expert clinician recommendations, this was expected. The expertise required to manage pediatric oral anticoagulation and the knowledge of individual children’s’ warfarin dose response is nuanced and improves with time and experience [36]. In practice, when the nomogram is used by a parent to support PSM, it will be done so in combination with support, education, and guidance from experienced clinicians [33]. The education includes advice for when the interval between tests must be shortened, such as when a change in diet, change in medication, or viral illness affects the child’s health status. Guidance documents that form part of the nomogram are provided to families during self-management education, detail the exact times to retest when a child experiences a change in health status. Furthermore, parents undertaking PSM or who are given the opportunity to use such a nomogram, do so after robust education and a lengthy period of clinician assisted dosing [33,36].

With the increased use of direct oral anticoagulants for thromboprophylaxis in children, there is a risk that as less children require warfarin, the expertise required to manage warfarin in children with complex underlying conditions, will decrease. Children with mechanical heart valves require lifelong anticoagulation and at present, warfarin remains the only oral anticoagulant that can provide optimal thromboprophylaxis [37]. Optimizing the management of children who do not have the option of a direct oral anticoagulant, such as those with mechanical heart valves remains a priority and the development of formalized warfarin dosing decision support tools, such as this nomogram, may supports patients, families, and clinicians.

This study was not without limitations. Missing data meant that there were more data points in some target INRs ranges compared with others and several individual children contributed significantly more warfarin dosing and INR retesting episodes to the study, which may have the capacity to influence the results. To overcome this, cluster analysis was performed to demonstrate the time in therapeutic range for each child and the median target range achievement for the sample [27]. Ongoing validation of the nomogram in more children with varying TTRs and unstable warfarin therapy is planned.

As a retrospective audit, some data were missing or unclear when comparing the nomogram to clinicians’ decisions due to inadequate documentation in Epic. Specifically, for INRs out of target range, there were instances where there was insufficient detail to determine if there was a recognized factor contributing to the clinician’s warfarin dosing or INR retesting decision which would align with advice from the nomogram. For example, clinicians recording a child was “unwell with cold” would be consistent with a corresponding factor in the nomogram such as “respiratory type of illness for more than 48 hours, fever, cough, lethargy, and runny nose with reduced appetite.” Further testing of this pediatric warfarin nomogram across a larger population is needed, as the highly selected inclusion criteria may limit capacity to extrapolate the results to a broader population of children at the tertiary hospital receiving long-term warfarin therapy, as suggested by Christensen [20]. To also explore how clinical factors affect the application of the nomogram, measurement of inter-user agreement is recommended in future studies. Measurement of inter-user agreement was not performed in this audit as it was beyond the scope of the study. Hence our understanding of the consistency in application of the nomogram across users is limited.

Additionally, ethnicity and race are not recorded as standard demographic data in the electronic medical records at our institution. We acknowledge that not including this information limits the ability to generalize the results to other samples of children requiring warfarin. However, as we are reporting a dosing tool rather than a medical intervention, we believe this limitation to be minor. This nomogram can be used to support warfarin self-management and may assist clinicians and patients or families to make evidence-based dosing decisions. In our experience, ethnicity and race are unlikely to influence warfarin dosing decisions.

## 5 Conclusions

The findings of this study demonstrate that this new pediatric warfarin nomogram is appropriate to guide dosing decisions for families engaged in PSM who have had extensive education, as indicated by agreement with specialized clinician warfarin dosing. Ongoing testing of the nomogram should be performed to further refine the recommendations for INR retesting based on the many factors influencing warfarin stability in children. The availability of a warfarin nomogram is crucial to support children and families to self-manage their warfarin dosing but may also be an important resource for clinicians practicing outside of specialized anticoagulation clinics or those with less experience in warfarin dosing in complex pediatric patients.
